# Congenital Clubfoot as an Early Manifestation of Duchenne Muscular Dystrophy?

**DOI:** 10.3390/muscles5030068

**Published:** 2026-09-19

**Authors:** Vladimir Kenis, Pavel Shpulev, Evgeniya Melnik, Irina Sosnina, Evgeniya Snegova

**Affiliations:** 1 H. Turner National Medical Research Center for Children’s Orthopedics and Trauma Surgery, Parkovaya 64–68, Pushkin, 196603 Saint Petersburg, Russia; 2 North-Western State Medical University named after I.I. Mechnikov, 191015 Saint Petersburg, Russia; 3 Research Centre for Medical Genetics, Moskvorechie Str., 1, 115522 Moscow, Russia; 4 Saint Petersburg State Budgetary Healthcare Institution “Consulting and Diagnostic Center for Children”, Aleksa Dundić Str., 36/2, 192289 Saint Petersburg, Russia

**Keywords:** congenital clubfoot, Duchenne muscular dystrophy, Ponseti method

## Abstract

Background: Duchenne muscular dystrophy (DMD) is the most common X-linked progressive neuromuscular disorder of childhood. The classic course of DMD is characterized by onset after a period of normal motor development. However, in recent years, evidence has been accumulating of prodromal features of the disease, including speech delays and neurocognitive impairments. Methods: This study investigates whether congenital bilateral clubfoot may represent the first clinical manifestation of DMD. Results: We present two cases of male patients with congenital bilateral clubfoot who were subsequently diagnosed with DMD. We demonstrate the high efficacy and safety of the Ponseti method for correcting foot deformities in these patients. We discuss a possible pathogenic link between dystrophin deficiency and impaired intrauterine myogenesis. Conclusions: This is the first report of an association between congenital clubfoot and DMD, highlighting the need for expanded early diagnostic protocols for boys presenting with idiopathic clubfoot.

## 1. Introduction

Congenital clubfoot (CC) is one of the most common congenital musculoskeletal anomalies requiring orthopedic intervention. The prevalence is estimated at 1–2 cases per 1000 live births, with a male-to-female ratio of approximately 2:1 [1]. Despite significant therapeutic advances achieved through widespread implementation of the Ponseti method [2,3], the etiology of CC remains poorly understood. Historically, the disease has been considered multifactorial, involving genetic, epigenetic, and environmental factors [4,5]. However, increasing attention has recently focused on the role of muscle tissue in the pathogenesis of this deformity, particularly on genes regulating the myofibrillar contractile apparatus [6,7].

Studies have shown that many candidate genes associated with idiopathic clubfoot are also implicated in congenital myopathies. This suggests that in some cases, what is termed ‘idiopathic’ clubfoot may represent a subclinical manifestation of systemic muscle pathology [8,9].

Duchenne muscular dystrophy (DMD, OMIM #310200) is a severe progressive disorder caused by mutations in the *DMD* gene, which encodes the dystrophin protein. The incidence is approximately 1 in 3500–5000 male births [10]. The traditional paradigm posits that muscle tissue in DMD undergoes degenerative changes primarily postnatally, when muscles begin to experience active mechanical loading. This results in pseudohypertrophy, followed by muscle weakness, contractures, and secondary orthopedic deformities [11]. The most common orthopedic manifestations during the ambulatory phase of DMD are progressive acquired equinovarus deformities of the feet, which eventually evolve into rigid neurogenic deformities [12].

To our knowledge, no reports have described congenital clubfoot in patients with a confirmed diagnosis of DMD.

This study aims to describe two cases of congenital clubfoot in children diagnosed with DMD, discuss possible common pathophysiological mechanisms underlying this association, and evaluate the effectiveness and safety of orthopedic treatment for congenital clubfoot in patients with DMD.

## 2. Results

We observed two patients with a confirmed diagnosis of DMD. Below, we describe the diagnosis, treatment, and outcomes following the Ponseti method for congenital clubfoot in these patients.

**Figure 1:**
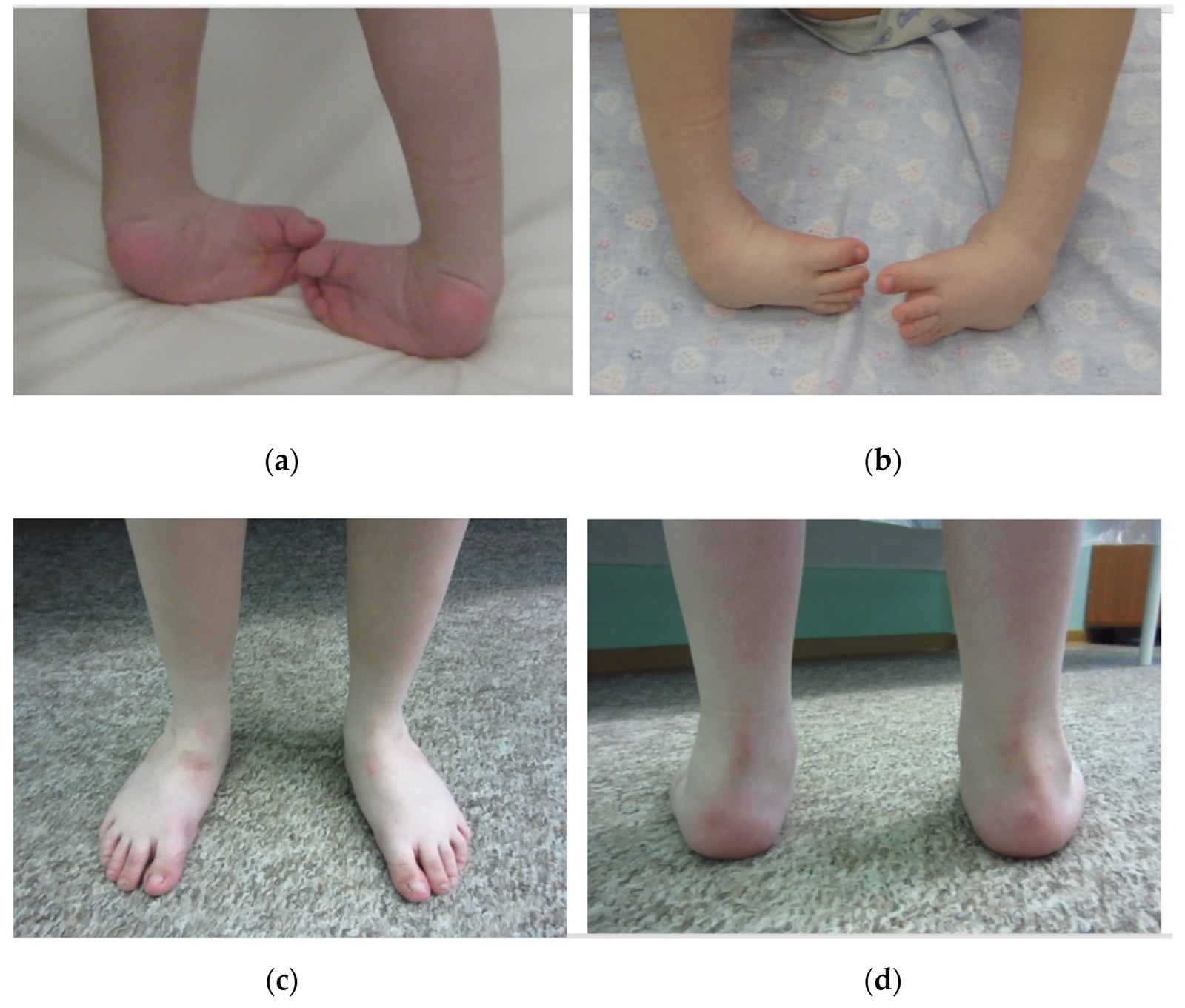
Clinical appearance of the feet of patient 1 before (**a**,**b**) and after (**c**,**d**) Ponseti treatment of late presented congenital clubfeet demonstrates excellent correction (plantigrade, normally aligned feet).

Patient 1, a boy, presented to our clinic at age 2.5 years. Bilateral congenital clubfoot deformity was diagnosed at birth. Serial plaster casting with short-leg casts was initiated at his primary care facility. This treatment was ineffective and complicated by adverse effects, including skin maceration, heel pressure ulcers, severe anxiety, and sleep disturbances. At 6 months of age, the parents were offered an extensive posteromedial release. Routine preoperative examination revealed markedly elevated serum creatine kinase (CK) levels—12,400 U/L (normal for age: <200 U/L). Moderately elevated transaminases (ALT 185 U/L, AST 210 U/L) were also noted. Although initially interpreted by pediatricians as hepatitis, subsequent liver ultrasound revealed no abnormalities. Liver pathology was excluded, and the hyperenzymemia was attributed to muscle cytolysis. Given the presence of congenital clubfoot, a neurologist suspected congenital myopathy; however, standard genetic testing for DMD was not performed (Figure 1a,b). The parents, who were psychologically unprepared to accept the genetic nature of the disease, insisted on surgical correction.

When the child presented to our clinic at age 2.5 years, we offered his parents comprehensive genetic evaluation and treatment using the Ponseti method. We conducted a multi-stage discussion with the parents, focusing on the risks of general anesthesia in the context of undiagnosed myopathy. As a compromise, we offered ‘test’ castings to assess tissue compliance. After observing impressive correction following just the first two stages (the foot abduction angle increased from −20° to +15°, and equinus decreased by 10°), the parents agreed to the full protocol. A total of seven casting stages were performed, achieving a foot abduction angle of 60°. Equinus remained at 110°, so bilateral subcutaneous Achilles tenotomy was performed under infiltration anesthesia with 1% lidocaine. Following tenotomy, passive dorsiflexion reached 15°. The child was immobilized in plaster casts for 4 weeks, then transitioned to an abduction brace (60° abduction, 10° dorsiflexion) worn at night until age 4.5 years. The correction outcome was considered excellent, with a Pirani score of 0, with mobile, painless, plantigrade feet (Figure 1c,d). However, the parents noted a delay in speech development: phrasal speech appeared after age 3, and elements of echolalia and socialization difficulties persisted, which were considered consistent with mild autism spectrum disorder. Fine motor skills were within the age norm.

At age 7, during a preschool checkup, his parents noted increased fatigue when climbing stairs. A positive Gower’s maneuver was noted, but pseudohypertrophy of the calf muscles was not evident (likely due to the clubfoot deformity). Genetic testing (MLPA) revealed an exon 45 deletion in the DMD gene, confirming the diagnosis. By age 9, gradual deterioration was noted: the 6 min walk test distance decreased to 280 m, and contractures appeared in the hip and knee joints. By age 11, the patient had lost the ability to ambulate independently and became wheelchair-dependent. Progressive right-sided thoracolumbar scoliosis with a Cobb angle of 40° developed, requiring orthotic management with a rigid Cheneau-type brace. A moderate equinovarus deformity developed (hindfoot varus approximately 20°, equinus 90°), which was interpreted not as a true relapse of clubfoot but rather as a typical neurogenic deformity secondary to muscle imbalance and loss of ambulation. Notably, the deformity did not progress to a degree requiring surgical intervention, possibly because of prolonged orthosis use at an early age. The patient demonstrated significant cognitive difficulties, including decreased auditory-verbal memory, attention deficit, and requiring tutoring at school.

Patient 2, a boy, presented to our orthopedic clinic at age 3 months. He was the product of his mother’s second pregnancy, which was complicated by threatened miscarriage in the first trimester. Delivery was at term via cesarean section. Birth weight was 3400 g, length 51 cm. The diagnosis of bilateral congenital clubfoot was established by a neonatologist immediately after birth. On examination by an orthopedist, the severity of the deformity was 6.0 on the Pirani scale bilaterally. In the neonatal period, prolonged icterus (bilirubin reached 280 μmol/L) required phototherapy. Laboratory examination revealed elevated transaminases (ALT 165 U/L, AST 178 U/L) and creatine kinase (CK 8900 U/L). Given the persistence of hyperenzymemia after bilirubin normalization, the neonatologist initiated a comprehensive biochemical and instrumental workup. Liver ultrasound, electrocardiography and cardiosonography revealed no abnormalities. Due to the boy’s elevated CK and as part of ongoing selective screening of newborns with hypercreatinekinemia, genetic testing was performed. MLPA detected a deletion of exons 53–62 in the DMD gene, resulting in a frameshift and being classified as pathogenic. The parents were informed of the diagnosis and received genetic counseling.

**Figure 2:**
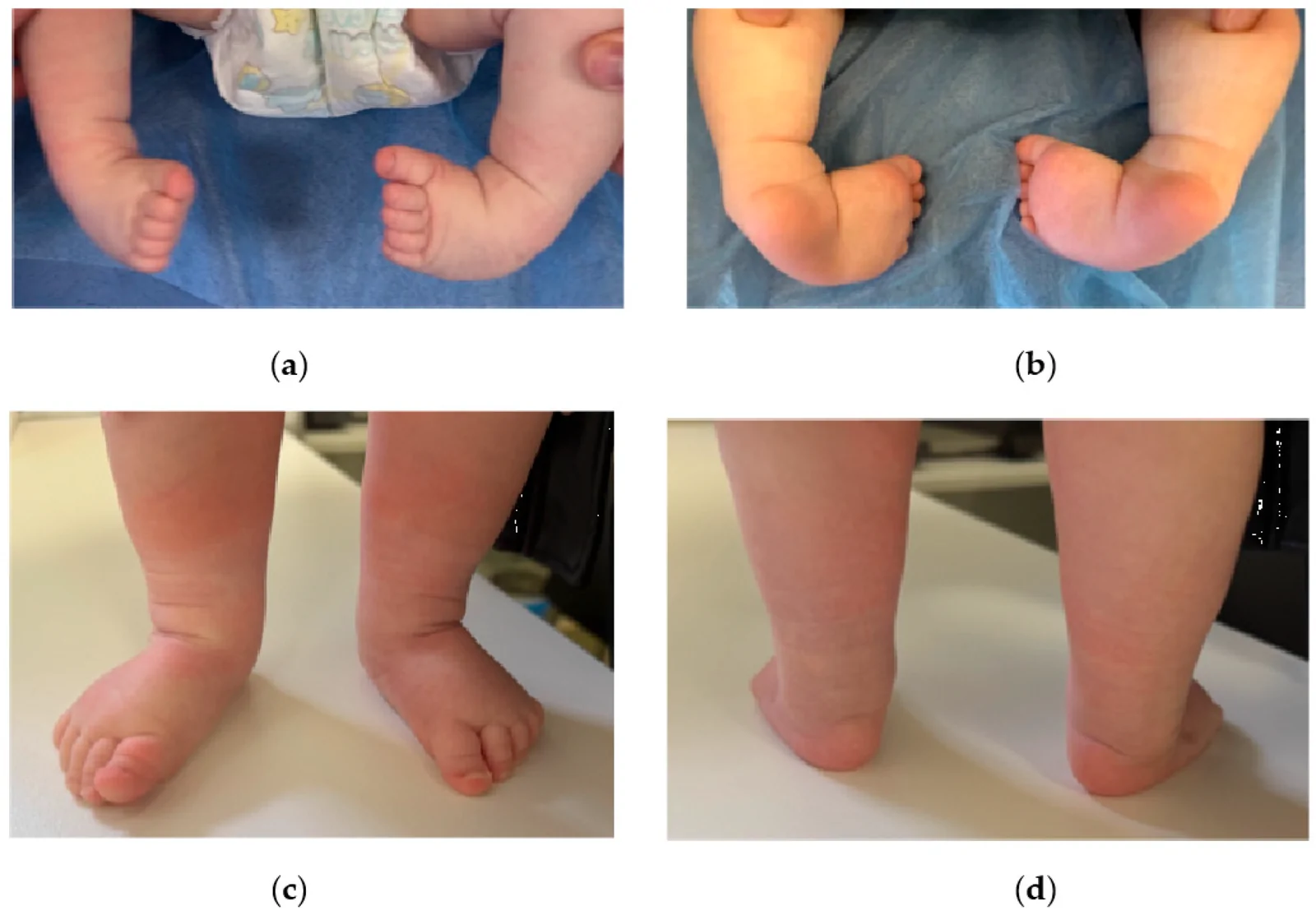
Clinical appearance of the feet of Patient 2 before (**a**,**b**) and after (**c**,**d**) Ponseti treatment of late presented congenital clubfeet demonstrates excellent correction (plantigrade, normally aligned feet).

At age 3 months, the child was referred to our clinic to establish a treatment strategy for clubfoot (Figure 2a,b). Based on the positive experience with the first patient and published data on the safety of the Ponseti method in patients with myopathies, we decided to begin the protocol immediately. The first casting stage was performed in a day-hospital setting and was well tolerated. A total of five casting stages were required. By the fourth stage, 60° of foot abduction was achieved, and equinus was reduced to 100°. At the final stage, under topical anesthesia (EMLA cream plus 1% lidocaine infiltration, 2 mg/kg), percutaneous Achilles tenotomy was performed bilaterally, achieving passive dorsiflexion of 15°. Standard abduction braces were applied and well tolerated. At 6 months, his feet remained fully corrected (Pirani score 0), he actively moved his toes, and bore weight on his entire foot. Currently (at 9 months), the boy continues to use the brace during daytime and nighttime sleep (Figure 2c,d).

## 3. Discussion

These cases are unique, as they represent the first documented association of congenital clubfoot with confirmed Duchenne muscular dystrophy. This finding prompts reconsideration of both the pathophysiological mechanisms of DMD and the diagnostic algorithms for congenital clubfoot. For many years, it has been axiomatic that muscular dystrophy due to dystrophin deficiency develops postnatally, because the ‘mechanical fragility’ of the sarcolemma is manifested only under load [13]. However, recent findings challenge this strict interpretation [14]. Dystrophin is expressed as early as the embryonic stages and is involved not only in maintaining membrane structural integrity but also in transmitting mechanotransduction signals that regulate myogenesis and neuromuscular synapse formation [15]. Congenital neuromuscular diseases often present with arthrogrypotic deformities and clubfoot, which are associated with impaired fetal motor activity (fetal akinesia) [16,17,18]. We hypothesize that certain severe DMD gene mutations causing critical dystrophin deficiency may impair lower leg muscle contractile function in utero, thereby triggering the formation of typical foot deformities.

In this regard, the compensatory mechanism involving utrophin deserves attention. Utrophin, encoded by the UTRN gene (localized at 6q24), is a structural and functional homologue of dystrophin. During embryonic development, utrophin is expressed throughout the sarcolemma and is later replaced by dystrophin as muscles mature, persisting in the adult organism primarily at the neuromuscular junction [19]. In DMD, utrophin represents the only endogenous protein capable of partially compensating for dystrophin deficiency. Important evidence linking utrophin to congenital contractures was recently reported in a study specifically examining the role of this protein in arthrogryposis [20]. The patient described in that study had severe bilateral congenital clubfoot. These findings suggest that common mechanisms involving the dystrophin/utrophin complex may manifest as equinovarus foot deformities.

The term ‘idiopathic congenital clubfoot’ has traditionally described an isolated foot deformity in a child without obvious signs of systemic disease. However, advances in molecular genetics and developmental biology have made it increasingly clear that this category is temporary and heterogeneous [21]. Dobbs and Gurnett (2012), in their seminal review, emphasized that a significant proportion of so-called idiopathic clubfoot cases may be attributable to subclinical neuromuscular disorders that manifest only during early embryogenesis and fetogenesis [22]. The authors noted that careful clinical and instrumental examination often reveals minimal signs of muscle hypotonia, electromyographic abnormalities, or ultrasound changes in the lower leg muscles in such patients. These data raise the possibility that what is commonly termed ‘idiopathic’ may actually represent a heterogeneous group encompassing a wide range of genetically determined myopathies that present with a single symptom. It is particularly noteworthy that even in the absence of obvious neurological symptoms in a newborn with clubfoot, muscle pathology should be excluded, as this has direct implications for prognosis and anesthetic management during surgical treatment.

Modifier genes may determine the degree of prenatal muscle weakness and consequently the risk of congenital contractures, as confirmed by arthrogryposis studies. This observation helps unravel the etiology of congenital clubfoot, suggesting that the ‘idiopathic’ form is more of a working classification that conceals a wide range of genetically determined muscle diseases [23,24].

Our cases of congenital clubfoot in Duchenne muscular dystrophy illustrate this concept well. Before the appearance of typical DMD features—such as proximal muscle weakness and calf pseudohypertrophy—the only visible abnormality in patient 1 was isolated bilateral clubfoot. Had it not been for the incidental discovery of hypercreatinekinemia, both patients might have been classified as having ‘idiopathic’ clubfoot for several years. This raises the possibility that among the thousands of children with this orthopedic diagnosis, many boys may have undiagnosed neuromuscular diseases, including mild allelic forms of DMD (Becker muscular dystrophy) or other late-onset conditions. Wider implementation of genetic screening (myopathy gene panels, exome sequencing) in newborns with clubfoot could fundamentally change our classification and enable detection of pathology long before irreversible changes develop. In this sense, the concept of ‘idiopathic’ clubfoot should be considered dynamic, and its boundaries will steadily narrow as diagnostic capabilities expand.

The empirical probability of a random co-occurrence of these two conditions is not zero. Given the incidence of DMD (1:4000) and congenital clubfoot (1:700–1:800) in boys, the estimated frequency of random co-occurrence is approximately 1 in 2–3 million. For a metropolitan area such as St. Petersburg (population approximately 5 million), random co-occurrence is possible but unlikely for two cases identified within a single clinic over a 10-year interval. For extremely rare hereditary diseases that occur de novo with comparable frequency—for example, fibrodysplasia ossificans progressiva (*ACVR1* gene)—the number of identified cases can be substantial [25], particularly given the presence of pronounced clinical manifestations [26]. Congenital clubfoot and DMD also have pronounced clinical presentations, although their manifestations are separated in time. We believe that congenital clubfoot in these patients represents a rare phenotypic manifestation of a systemic process rather than a simple coincidence.

Both cases clearly demonstrate that the Ponseti method is a safe and highly effective tool for correcting clubfoot, even in the presence of severe comorbid neurological pathology. The utilization of myography in future studies, as well as monitoring the distribution of foot pressure before and after the correction, will help make the corrections as accurate and effective as possible.

In patients with DMD (including those undiagnosed), the use of muscle relaxants and inhaled anesthetics carries a risk of malignant hyperthermia and acute rhabdomyolysis with hyperkalemia, which can lead to cardiac arrest [27,28,29,30]. Percutaneous Achilles tenotomy under local infiltration or topical anesthesia, performed as part of the Ponseti protocol, completely avoids this risk during equinus correction.

Non-idiopathic forms of clubfoot demonstrate a more severe course, lower responsiveness to standard Ponseti treatment, and a higher relapse rate [31]. The casting course in both our patients did not differ from idiopathic cases, either in the number of casts required or in the outcomes achieved. This confirms that in the early stages, the elasticity of foot tissues in DMD patients is sufficient for standard manipulation. The subsequent development of equinovarus deformity in the first case followed the natural history of the underlying neuromuscular disease (muscle imbalance and contracture) and was not a relapse of the primary deformity. Notably, even years after completion of the active treatment phase, the first patient did not require repeat foot surgery, which favorably distinguishes the Ponseti method from traditional posteromedial release—the results of which are often unsatisfactory in patients with neuromuscular diseases due to scarring and muscle weakness.

In both cases, the key marker that raised suspicion of muscle pathology was a marked elevation in CK. Congenital clubfoot combined with hyperenzymemia in a boy should first and foremost prompt exclusion of DMD and other muscular dystrophies, rather than being attributed to perinatal liver damage or nonspecific cytolysis. Given the availability of targeted therapies [32,33,34] and the need to initiate steroid therapy [35,36], the delay in genetic verification observed in the first case is now unacceptable [37,38]. Therapy for rare hereditary diseases is now quite diverse [39,40] and is not limited exclusively to targeting causative factors—parallel areas of symptomatic therapy are also developing, for which an accurate diagnosis and understanding of pathogenesis are equally important [41,42,43]. This is particularly relevant in light of the introduction of selective screening for DMD in newborn boys with elevated CK levels—similar to that which enabled early diagnosis in the second patient. Timely diagnosis freed the family from prolonged uncertainty and allowed planning of disease-modifying treatment.

## 4. Conclusions

These two cases of bilateral congenital clubfoot in boys with Duchenne muscular dystrophy represent the first documented description of this association in the literature. Despite our proposal of the uniquity of our cases, this statement should be treated with caution, as the sample size is minimal. Muscle pathology caused by dystrophin deficiency may, in some cases, disrupt normal intrauterine myogenesis and fetal motor activity, manifesting as orthopedic deformity long before the typical clinical picture of myopathy emerges. Clinicians—orthopedists and neonatologists alike—should exercise caution: the detection of persistent hyperenzymemia in a boy with clubfoot should prompt immediate exclusion of Duchenne muscular dystrophy. The Ponseti method has demonstrated impressive efficacy and a favorable risk profile in treating these complex patients, avoiding general anesthesia and major surgical interventions. Further study of the role of dystrophin and its modifiers may open new horizons for predicting and preventing orthopedic complications.

## Data Availability

The original contributions presented in this study are included in the article. Further inquiries can be directed to the corresponding author.

## Abbreviations

The following abbreviations are used in this manuscript:

CC	Congenital Clubfoot
DMD	Duchenne Muscular Dystrophy
CK	Creatine kinase
MLPA	Multiplex ligation-dependent probe amplification
ALT	Alanine aminotransferase
AST	Aspartate aminotransferase
